# Dose-Limiting Organs at Risk in Carbon Ion Re-Irradiation of Head and Neck Malignancies: An Individual Risk-Benefit Tradeoff

**DOI:** 10.3390/cancers11122016

**Published:** 2019-12-13

**Authors:** Thomas Held, Semi B. Harrabi, Kristin Lang, Sati Akbaba, Paul Windisch, Denise Bernhardt, Stefan Rieken, Klaus Herfarth, Jürgen Debus, Sebastian Adeberg

**Affiliations:** 1 Heidelberg University Hospital, Department of Radiation Oncology, 69120 Heidelberg, Germany; thomas.held@med.uni-heidelberg.de (T.H.); semi.harrabi@med.uni-heidelberg.de (S.B.H.); sati.akbaba@med.uni-heidelberg.de (S.A.); denise.bernhardt@med.uni-heidelberg.de (D.B.); stefan.rieken@med.uni-heidelberg.de (S.R.); klaus.herfarth@med.uni-heidelberg.de (K.H.);; 2 Heidelberg Institute of Radiation Oncology (HIRO), 69120 Heidelberg, Germany; 3 National Center for Tumor diseases (NCT), 69120 Heidelberg, Germany; 4 Heidelberg Ion-Beam Therapy Center (HIT), 69120 Heidelberg, Germany; 5 University Hospital of Zurich, Department of Radiation Oncology, 8091 Zurich, Switzerland; 6 Clinical Cooperation Unit Radiation Oncology, German Cancer Research Center (DKFZ), 69120 Heidelberg, Germany; 7 German Cancer Consortium (DKTK), partner site Heidelberg, German Cancer Research Center (DKFZ), 69120 Heidelberg, Germany

**Keywords:** head and neck cancer, local recurrence, planning target volume, heavy ion therapy, dose constraints

## Abstract

*Background*: Carbon ion re-irradiation (CIR) was evaluated to investigate treatment planning and the consequences of individual risk-benefit evaluations concerning dose-limiting organs at risk (OAR). *Methods*: A total of 115 consecutive patients with recurrent head and neck cancer (HNC) were analyzed after initial radiotherapy and CIR at the same anatomical site. Toxicities were evaluated in line with the Common Terminology Criteria for Adverse Events 4.03. *Results*: The median maximum cumulative equivalent doses applied in fractions of 2 Gy (EQD2) to the brainstem, optic chiasm, ipsilateral optic nerve, and spinal cord were 56.8 Gy (range 0.94–103.9), 51.4 Gy (range 0–120.3 Gy), 63.6 Gy (range 0–146.1 Gy), and 28.8 Gy (range 0.2–87.7 Gy). The median follow up after CIR was 24.0 months (range 2.5–72.0 months). The cumulative rates of acute and late severe (≥grade III) side effects after CIR were 1.8% and 14.3%. *Conclusion*: In recurrent HNC, an individual risk-benefit tradeoff is frequently inevitable due to unfavorable location of tumors in close proximity to vital OAR. There are uncertainties about the dose tolerance of OAR after CIR, which warrant increased awareness about the potential treatment toxicity and further studies on heavy ion re-irradiation.

## 1. Introduction

Re-irradiation in cases of head and neck cancer (HNC) is associated with an increased risk of severe side effects [1,2,3]. The total cumulative applied radiation dose, the irradiated volume [4], and the time interval [5,6,7] are crucial when considering a second course of irradiation at the same anatomical site. Nonetheless, toxicity rates are frequently underestimated, even in prospective clinical studies [8]. Other factors that influence the dose tolerance of organs at risk (OAR) are fractionation effects [9] and the simultaneous administration of systemic therapies [10]. In addition, location effects impact the probabilities of normal tissue complications since radiosensitive structures are not homogeneously distributed within organs [11]. Patient related co-factors such as smoking or age may increase the rate of side effects. Many uncertainties remain in regard to the effects of dose volume on the probabilities of complications of OAR after re-irradiation [12,13,14]. 

The high linear energy transfer of carbon ion radiotherapy results in greater relative biological effectiveness (RBE) than photons, which depends on the irradiated tissue [15]. Re-irradiation with heavy ions enables a favorable depth–dose distribution, which allows for escalation of the dose in a recurrent tumor while avoiding normal surrounding tissue and OAR [16,17]. Limiting the total dose applied to OAR is essential for preventing severe side effects, such as vision loss. 

Data on carbon ion re-irradiation (CIR) are scarce, and there are currently no guidelines for dose constraints regarding OAR for the treatment of recurrent HNC with carbon ions. Uncertainties regarding the dose tolerance of normal tissue after heavy ion re-irradiation necessitate greater awareness of treatment-specific toxicities. Therefore, CIR was analyzed to investigate treatment planning and the effects of individual risk–benefit evaluations concerning OAR.

## 2. Results

### 2.1. Patient Characteristics

The most common tumor site was the paranasal sinuses (*n* = 33, 28.7%), followed by the salivary glands (*n* = 19, 16.5%) and the nasopharynx (*n* = 22, 19.1%). The vast majority of patients had adenoid cystic carcinomas (ACC, *n* = 77, 67.0%) or squamous cell carcinomas (SCC, *n* = 23, 20.0%). Prior to CIR, staging was conducted according to the 8th edition of the Union for International Cancer Control (UICC) tumor node metastasis (TNM) system. Tumor infiltration of the base of the skull or the immediate surroundings occurred in 94 patients (*n* = 94, 81.7%). Detailed patient and tumor characteristics are shown in Table 1.

### 2.2. Radiation Therapy

#### 2.2.1. Initial Radiotherapy

The first radiation treatment consisted of 3D-conformal RT in 8 patients (7.0%), intensity-modulated radiation therapy (IMRT) in 39 patients (33.9%), and bimodal RT (IMRT and carbon ion boost) in 60 patients (52.2%). A total of six patients (*n* = 6, 5.2%) received carbon ion radiotherapy as initial treatment. The irradiation technique was unspecified in two patients (1.7%). Patients with bimodal RT received a median dose of 50 Gy (range 48–56 Gy) with photon IMRT and a median dose of 24 Gy (RBE) (range 18–24 Gy (RBE)) with carbon ions. A total of seven patients (*n* = 7, 6.1%) received two prior irradiations at the same anatomical site.

#### 2.2.2. Carbon Ion Re-Irradiation

The median interval between the previous course of irradiation and CIR was 3.4 years (range 0.3–13.3 years). A median total dose of 51 Gy (RBE) (range 39–60 Gy (RBE)) in fractions of 3 Gy (RBE) was applied. The median PTV and CTV were 128.9 cc (range 13.3–925.0 cc) and 83.1 cc (range 6.3–710.5 cc), respectively. No simultaneous systemic therapy was applied during CIR. The equivalent doses in 2-Gy fractions (EQD2) were calculated with an assumed α/β of 2 for ACC and 10 for SCC and other tumor entities according to the local effect model (LEM I). The median cumulative EQD2 after initial irradiation and CIR was 135.8 Gy (range 101.3–155.5 Gy). Detailed information about the initial RT and CIR are shown in Table 2.

#### 2.2.3. Organs at Risk

For the optic nerves, temporal lobes, and mandibular joints, only the total dose to the ipsilateral side was considered for lateralized tumors. The median maximum cumulative EQD2 applied to the brainstem, optic chiasm, ipsilateral optic nerve, and spinal cord was 56.8 Gy (range 0.94–103.9), 51.4 Gy (range 0–120.3 Gy), 63.6 Gy (range 0–146.1 Gy), and 28.8 Gy (range 0.2–87.7 Gy), respectively. Information about the irradiated volume of the respective OAR was not specified. 

Generally, the maximum applied dose was limited to the surface area. However, in 49 patients (*n* = 49, 42.6%), the orbital cavity was infiltrated by the tumor. Consequently, the maximum dose constraint for the ipsilateral optic nerve was disregarded in individual cases upon patient consent to achieve improved tumor control. In these patients, the median maximum cumulative EQD2 applied to the ipsilateral optic nerve and optic chiasm was 113.1 Gy (range 65.5–146.1 Gy) and 66.8 Gy (range 14.5–120.3 Gy), respectively. 

When these cases were excluded (*n* = 66 patients, 57.4%), the maximum cumulative EQD2 applied to the ipsilateral optic nerve and optic chiasm was 42.4 Gy (range 0–102.7 Gy) and 27.4 Gy (range 0–93.6 Gy), respectively. The total dose of CIR applied to the brainstem, optic nerve, and spinal cord was 20% higher if the RT interval was more than two years. Further information on the total dose applied to OAR is shown in Table 3.

### 2.3. Acute and Late Toxicity

The median follow up after CIR was 24.0 months (range 2.5–72.0 months). Acute toxicities included new or worsening adverse events that were attributable to CIR and occurred from the start until 90 days after radiation treatment. All patients completed re-irradiation without interruptions. The cumulative rate of acute severe toxicity (≥grade III) was 1.8%. There were no cases of grade IV or V acute toxicity. Low grades of acute toxicity (grades I–II) most commonly included radiation dermatitis, oral mucositis, and xerostomia. Several patients developed hearing impairment due to unilateral middle-ear inflammation. Furthermore, neurological side effects such as trigeminal nerve disorder were common. 

Late toxicity was observed in 70 patients (60.9%) with available long-term clinical follow up and imaging. The cumulative rate of late severe toxicity was 14.3%. High-grade (≥grade III) optic nerve disorder occurred in four patients. In two of these patients, the orbital cavity was infiltrated by the tumor. After obtaining patient consent, the maximum dose constraint for the ipsilateral optic nerve was disregarded to increase the tumor control rate. The time intervals from the start of CIR until the occurrence of high-grade optic nerve disorder in these patients were 2.4 and 16.3 months for grade III and 7.7 and 40.1 months for grade IV, respectively. 

In six patients (5.2%), an enucleation of the eye was performed prior to CIR due to tumor infiltration. Grade II and III necrosis of the central nervous system (CNS) occurred in 9 (7.8%) and 4 (3.5%) patients, respectively. The management of CNS necrosis after CIR is beyond the scope of this study [3]. 

Dose limits for the optic nerves and temporal lobes were surpassed in all of the patients with severe late (≥grade III) optic nerve disorder (*n* = 4) and CNS necrosis (*n* = 4). In addition, all patients with a maximum cumulative dose beyond the constraints [18] of 54 Gy for the optic system *(n* = 39), 60 Gy for the temporal lobe (*n* = 22), and long-term clinical follow up and imaging were analyzed. Of these patients, 10.3% (*n* = 4) and 18.2% (*n* = 4) developed optic nerve disorder and CNS necrosis, respectively. Data on acute and late toxicity are shown in Table 4.

## 3. Discussion

Patients with recurrent HNC frequently received a total cumulative dose that was above the maximum dose constraints for OAR such as the temporal lobe, depending on the radiotherapy interval. In these cases, a risk–benefit trade off was inevitable due to an unfavorable tumor location near OAR. Nonetheless, the cumulative rate of acute and late severe toxicity of 14.3% was acceptable. In most patients with high-grade (≥ grade III) late toxicity, the dose limits were surpassed. 

In patients with tumors infiltrating the orbital cavity (*n* = 49, 42.6%), dose constraints for the ipsilateral optic nerve were disregarded upon patient consent to achieve improved tumor control. Of these patients, 10.3% (*n* = 4) developed optic nerve disorder (≥grade III). Considering the relatively short median follow up of 24.0 months, the expected rate of late adverse events will probably increase over time in these patients. Contrary to the assumptions of treatment planning, the exact dose maxima of the initial RT and CIR were not entirely congruent in the current analysis. Therefore, dose–volume effects were probably equally critical for preventing treatment toxicity. In patients without orbital cavity infiltration, the median maximum cumulative EQD2 applied to the ipsilateral optic nerve (42.4 Gy) was within dose limits. 

The RT interval is another substantial factor that impacts the risk–benefit evaluation prior to CIR [19,20]. The total dose of CIR applied to OAR was 20% higher if the minimum RT interval was more than two years. Considering all patients for whom dose constraints were exceeded intentionally (*n* = 49, 42.6%), the dose prescription at CIR was restrictive in the current analysis. Hayashi et al. [21] reported absolute dose constraints at CIR of 30 Gy (RBE) for the brainstem and spinal cord and 40 Gy (RBE) for the optic system. Hu et al. [6] assumed a recovery from the previous photon radiotherapy set at 70%, independent of the RT interval. Consequently, the CTV was reduced if it was close to OAR. However, the irradiated volume of the respective OAR was not specified in the current evaluation. 

Currently, there are no guidelines regarding the dose tolerance of OAR after heavy ion re-irradiation. Therefore, a thorough risk–benefit evaluation is required for each individual patient. The potential for severe early and late side effects after CIR depends primarily on the location of the tumor. In particular, re-irradiation of tumors that have infiltrated the base of the skull result in higher doses applied to dose-limiting OAR, such as the brainstem and temporal lobes [22]. Furthermore, simultaneous systemic therapies lead to a significant increase in treatment-related toxicities [1,2]. However, in prior evaluations on heavy-ion re-irradiation [3,6,23], concomitant systemic therapies were primarily excluded. The clinical effects of CIR in combination with systemic agents have yet to be evaluated. 

According to this current data analysis and guided by the clinical experience in CIR of recurrent HNC at our institution since 2010, several constraints of dose-limiting OAR can be pointed out. If the RT interval is less or equal to two years, the proposed cumulative radiation tolerance of the optic system and brain stem complies to the QUANTEC [13] analyses. However, if the RT interval is more than two years, an increase of the total cumulative dose to OAR by up to 20% has shown acceptable toxicity profiles [20]. Based on these results, proposed dose limits for CIR are provided in Table 5. Individual dose prescription can differ and will be at the discretion of the radiation oncologist.

The time intervals until the occurrence of high-grade toxicities after CIR varied considerably. The onset of optic nerve disorders determined from the start of CIR ranged from 2.4–40.1 months. Possible explanations for this finding include the impact of volume and location effects, as well as the total dose applied in the initial radiation treatment. In contrast, the onset of CNS necrosis after CIR followed a less elusive pattern. Most tumors infiltrated the base of the skull, so the median maximum dose of 52.8 Gy (RBE) (range 0–64.0 Gy (RBE)) applied to the temporal lobe at CIR was extensive. Grades I, II, and III CNS necrosis occurred after median time intervals of 8.8 (range 5.5–75.0 months), 8.2 (range 2.3–25.5 months), and 14.2 months (range 8.7–32.5 months), respectively. The clinical outcomes with early and adequate treatment were encouraging [3]. 

The cumulative rates of acute and late severe (≥ grade III) side effects after CIR were acceptable at 1.8% and 14.3%, respectively. Prior studies on re-irradiation of HNC with carbon ions reported similar toxicity profiles [6,23,24]. However, the distribution of re-irradiation sites in our assessment was quite different from those of prior studies on CIR in recurrent HNC. In this study, 49 patients (42.6%) presented with tumor infiltration of the orbital cavity, and 94 patients (81.7%) had tumors with infiltration of the base of the skull or the immediate surroundings. Furthermore, compared to prior studies on re-irradiation with heavy ions [21], the median PTV of 128.9 cc in this evaluation was extensive, reaching about 50–100% more. In addition, the tumor locations were possibly more challenging in the current analysis (81.7% of them infiltrated the base of the skull).

This analysis had several limitations worth mentioning. First, the rates of treatment toxicities could have been underestimated due to the retrospective design of the study. In a prospective setting, side effects related to CIR might be reported more frequently. Particularly, long-term sequelae and chronic late radiation effects were underrated since several patients were lost to follow up due to the poor prognosis. Second, the heterogeneity of the initial radiation treatment limits the validity of our results. Third, only the total dose applied to OAR was recorded, and volume effects were not considered. In addition, differences remain in the fractionation and uncertainties of the RBE of carbon ions in the clinical setting. 

Despite the limitations, the analysis was independently validated by several experts in the field of heavy ion therapy. Furthermore, a comparatively large patient cohort (*n* = 115) was analyzed in regard to dose-limiting OAR, considering the scarcity of data on CIR in recurrent head-and-neck malignancies. To evaluate CIR in recurrent HNC further, a treatment plan comparison including photon and proton plans is currently being prepared.

## 4. Materials and Methods 

### 4.1. Patient Characteristics

After approval by the local ethics committee (S-421/2015), patients from the cancer database of the National Center for Tumor Diseases (NCT) with head and neck cancer (HNC) were screened. All patients with at least one prior course of radiotherapy (RT) and CIR at the same anatomical site were evaluated. Patients without complete information on the total cumulative dose applied to the optic system, brainstem, and spinal cord were excluded from this retrospective study. A total of 115 (*n* = 115) patients with HNC treated at our clinic with CIR between 2010 and 2018 were analyzed, and 10 patients (*n* = 10, 8.7%) received surgical resection prior to CIR.

### 4.2. Treatment Planning

Patients were immobilized with a thermoplastic head mask. Computed tomography (CT) scans (3-mm slice thickness) were used for treatment planning, and contrast-enhanced T1-weighted magnetic resonance imaging (MRI) was used for image registration. Treatment planning was conducted using Syngo PT Planning version 13 (Siemens^®^, Erlangen, Germany). The clinical target volume (CTV) included the gross tumor volume in contrast-enhanced CT or MRI with an additional margin of 2–5 mm. In patients with surgical resection prior to CIR, the resection cavity was included in the CTV. No elective lymph node treatment was performed. A safety margin of 2–3 mm was added for the planning target volume (PTV), depending on the patient positioning and beam arrangement. 

Prior to CIR, the cumulative dose to be applied was determined for OAR (the optic system, brainstem, and spinal cord). To account for differences in fractionation, an assumed α/β of 2 was used for the brainstem, temporal lobe, and spinal cord. For the optic system and mandibular joint, α/β values of 3 and 3.5 were used for dose calculation. In individual cases, the CTV was adapted by the treating radiation oncologist to reduce the cumulative dose applied to vital OAR. Treatment was performed at our clinic using the active raster scanning method with daily image guidance by orthogonal X-rays, as well as routine CT scans and position correction in six degrees of freedom.

### 4.3. Follow Up

Imaging included contrast-enhanced MRI or CT scans of the head and neck, which were scheduled six to eight weeks after CIR and then every three months thereafter within the first year after treatment. Symptoms and toxicities related to treatment were recorded in a non-standardized manner by a clinician at each follow-up visit. Patients also presented regularly to an ear, nose, and throat specialist or oral and maxillofacial specialist for clinical examination.

### 4.4. Statistics

Statistical analyses were conducted using SPSS Statistics 25 (IBM^®^, New York, USA) and the software R version 3.4.3 (www.r-project.org). The median follow-up was calculated using the inverse Kaplan-Meier method. Toxicities were evaluated from the patients’ medical records in line with the Common Terminology Criteria for Adverse Events (CTCAE) 4.03.

## 5. Conclusions

In recurrent HNC, a risk–benefit tradeoff is frequently inevitable due to an unfavorable location of the tumor near OAR. Dose constraints may be exceeded in individual dose prescriptions in CIR to improve local tumor control after obtaining patient consent. Since there are uncertainties about the dose tolerance of OAR after CIR, increased awareness for potential treatment toxicity and further studies on heavy ion re-irradiation are necessary.

## Figures and Tables

**Table 1:** Patient characteristics (*n* = 115 patients).

Characteristics	Patients	%
Female/Male	53/62	46.1/53.9
ECOG status		
0	61	53.0
1	51	44.4
2	3	2.6
Histology		
ACC	77	67.0
HNSCC	23	20.0
Adenocarcinoma	9	7.8
Other	6	5.2
Tumor Site		
Paranasal sinuses	33	28.7
Nasopharynx	22	19.1
Salivary glands	19	16.5
Skull base	13	11.3
Oral cavity	10	8.7
Oropharynx	8	7.0
Other	10	8.7
TNM stage prior to CIR		
T1-2	7	6.1
T3-4	89	77.4
TX	19	16.5
N0	81	70.4
N1	3	2.6
N2	13	11.3
NX	18	15.7
M0	89	77.4
M1	26	22.6

Abbreviations: Eastern Cooperative Oncology Group (ECOG), adenoid cystic carcinoma (ACC), head and neck squamous cell carcinoma (HNSCC), tumor node metastasis (TNM), carbon ion re-irradiation (CIR).

**Table 2:** Treatment characteristics (*n* = 115 patients).

Characteristics	Patients	%
Initial RT		
Bimodal RT	60	52.2
IMRT	39	33.9
3DCRT	8	7.0
CIRT	6	5.2
Unspecified	2	1.7
Salvage surgery prior to re-irradiation		
Yes	10	8.7
No	105	91.3
	Median	Range
Carbon ion re-irradiation		
Total dose [Gy (RBE)]	51.0	39.0–60.0
Total dose [EQD2]	63.8	48.8–75.0
Fractions	17	13–20
Planning target volume	128.9	13.3–925.0
Clinical target volume	83.1	6.3–710.5
Further treatment parameters		
Cumulative Dose [EQD2]	135.8	101.3–155.0
RT-Interval [years]	3.4	0.3–13.3
Prior tumor-specific treatments	2	1–8

Abbreviations: Radiotherapy (RT), intensity modulated radiation therapy (IMRT), IMRT and carbon ion boost (bimodal RT), 3D conformal radiation therapy (3DCRT), carbon ion radiotherapy (CIRT), equivalent dose in 2 Gy fractions (EQD2), relative biological effectiveness (RBE).

**Table 3:** Organs at risk (*n* = 115 patients).

OAR	Initial RT [EQD2]		CIR [Gy (RBE)]		CIR [EQD2]		Initial RT+CIR [EQD2]	
	Median	Range	Median	Range	Median	Range	Median	Range
**Mean**								
Brainstem	16.6	0–39.8	1.5	0–16.6	0.8	0–12.1	17.7	0–46.3
Optic chiasm	14.3	0–53.8	3.2	0–46.0	2.0	0–52.5	18.4	0–87.3
Optic nerve	26.3	0–74.0	12.8	0–57.1	9.5	0–68.6	38.4	0–124.0
Optic nerve *	8.4	0–66.7	0.7	0–38.2	0.4	0–40.1	15.2	0–68.1
Brain	n.a.	n.a.	9.8	0–29.4	6.3	0–27.4	n.a.	n.a.
Mandibular joint	n.a.	n.a.	27.0	0–59.6	25.2	0–67.0	n.a.	n.a.
Spinal cord	9.3	0–48.0	0.1	0–4.2	0.1	0–2.3	9.3	0–49.5
**Maximum**								
Brainstem	45.6	0–67.8	19.7	0–49.8	15.6	0–59.3	56.8	0.9–103.9
Optic chiasm	30.7	0–60.2	17.3	0–56.1	14.1	0–70.7	51.4	0–120.3
Optic nerve	46.2	0–76.5	28.5	0–64.7	26.4	0–83.0	63.3	0–146.1
Optic nerve *	21.4	0–71.0	6.6	0–50.9	4.4	0–61.1	42.4	0–102.7
Brain	n.a.	n.a.	52.8	0–64.0	67.6	0–88.9	n.a.	n.a.
Mandibular joint	n.a.	n.a.	43.1	0–59.6	50.2	0–72.0	n.a.	n.a.
Spinal cord	27.8	0–52.3	1.2	0–53.8	0.6	0–69.6	28.8	0.2–87.7

Abbreviations: organ at risk (OAR), Radiotherapy (RT), carbon ion re-irradiation (CIR), equivalent dose in 2 Gy fractions (EQD2), * excluding 49 patients with tumors infiltrating the orbital cavity.

**Table 4:** Acute and late treatment related toxicity.

Toxicity	*n*=	%
Acute toxicity		
*High grade (CTCAE III–IV)*		
Dysphagia °III	1	0.9
Hearing impairment °III	1	0.9
*Low grade (CTCAE I–II)*		
Dermatitis radiation °I–II	38	33.1
Mucositis oral °I–II	30	26.1
Xerostomia °I–II	23	20.0
Headache °I–II	14	12.1
Alopecia °I	11	9.6
Hearing impairment °I–II	11	9.5
Dysgeusia °I–II	10	8.7
Trismus °I	10	8.7
Dysphagia °I–II	9	7.8
Middle ear inflammation °I–II	9	7.8
Conjunctivitis °I–II	6	5.2
Weight loss °I–II	4	3.5
Trigeminal nerve disorder °I–II	3	2.8
Optic nerve disorder °I–II	3	2.8
**Late toxicity**		
*High grade (CTCAE III–IV)*		
Optic nerve disorder °III–IV	4	5.7
Hearing impairment °III	2	2.9
CNS necrosis °III	4	5.7
*Low grade (CTCAE I–II)*		
CNS necrosis °I–II	21	30
Xerostomia °I–II	14	20.0
Hearing impairment °I–II	12	17.1
Trismus °I	10	14.3
Dysgeusia °I–II	10	14.5
Middle ear inflammation °I–II	9	12.9
Headache °I–II	7	10.0
Trigeminal nerve disorder °I–II	7	10.0
Dysphagia °I–II	5	7.2
Alopecia °I	5	7.1
Weight loss °I–II	3	4.3
Osteonecrosis °I–II	3	4.3
Optic nerve disorder °I–II	2	2.9

Abbreviations: Common Terminology Criteria for Adverse Events (CTCAE), central nervous system (CNS).

**Table 5:** Proposed radiation tolerance of organs at risk for carbon ion re-irradiation.

OAR	Maximum Cumulative BED2Gy(RT interval ≤ 2 years)	Maximum Cumulative BED2Gy(RT interval > 2 years)	Comment
Brain stem *(α/β = 2)*	60	72 (≙+20%)	Maximum (surface)
Optic chiasm *(α/β = 3)*	54	64.8 (≙+20%)	Maximum
Optic nerves *(α/β = 3)*	54	64.8 (≙+20%)	Maximum
Spinal cord *(α/β = 2)*	50	60 (≙+20%)	Maximum
Jawbone, inner ear, skin, mandibular joint, mandibular bone, lacrimal gland, parotid gland, submandibular gland, eyes, internal carotid arteries, temporal lobes	ALARA *		/

Abbreviations: organ at risk (OAR), biological effective dose in 2 Gy fractions (BED2Gy), radiotherapy (RT), as low as reasonably achievable (ALARA).
